# COVID-19: Underlying Adipokine Storm and Angiotensin 1-7 Umbrella

**DOI:** 10.3389/fimmu.2020.01714

**Published:** 2020-07-21

**Authors:** Geoffroy Méry, Olivier Epaulard, Anne-Laure Borel, Bertrand Toussaint, Audrey Le Gouellec

**Affiliations:** ^1^Service Hospitalier Universitaire de Pneumologie Physiologie, CHU Grenoble-Alpes, La Tronche, France; ^2^Service de Maladies Infectieuses et Tropicales, CHU Grenoble-Alpes, La Tronche, France; ^3^Groupe de Recherche en Infectiologie Clinique, Université Grenoble Alpes, La Tronche, France; ^4^UMR 5075—Institut de Biologie Structurale, Grenoble, France; ^5^Service de Nutrition, Pole DIGIDUNE, CHU Grenoble-Alpes, La Tronche, France; ^6^Hypoxia PathoPhysiology Laboratory, INSERM U1042, University Grenoble Alpes, La Tronche, France; ^7^Laboratoire TIMC-TheREx UMR 5525 CNRS-Université Grenoble Alpes, La Tronche, France; ^8^Service de Biochimie Biologie Moléculaire Toxicologie Environnementale, UM Biochimie des Enzymes et des Protéines, Institut de Biologie et Pathologie, C.H.U. Grenoble-Alpes, La Tronche, France

**Keywords:** coronavirus, SARS-CoV, ACE2, obesity, adipocyte, metabolic syndrome, inflammation

## Abstract

Severe acute respiratory syndrome coronavirus 2 (SARS-CoV-2) is the third coronavirus leading to a global health outbreak. Despite the high mortality rates from SARS-CoV-1 and Middle-East respiratory syndrome (MERS)-CoV infections, which both sparked the interest of the scientific community, the underlying physiopathology of the SARS-CoV-2 infection, remains partially unclear. SARS-CoV-2 shares similar features with SARS-CoV-1, notably the use of the angiotensin conversion enzyme 2 (ACE2) as a receptor to enter the host cells. However, some features of the SARS-CoV-2 pandemic are unique. In this work, we focus on the association between obesity, metabolic syndrome, and type 2 diabetes on the one hand, and the severity of COVID-19 infection on the other, as it seems greater in these patients. We discuss how adipocyte dysfunction leads to a specific immune environment that predisposes obese patients to respiratory failure during COVID-19. We also hypothesize that an ACE2-cleaved protein, angiotensin 1-7, has a beneficial action on immune deregulation and that its low expression during the SARS-CoV-2 infection could explain the severity of infection. This introduces angiotensin 1-7 as a potential candidate of interest in therapeutic research on CoV infections.

## Introduction

Coronavirus (CoV) is a single-stranded RNA virus involved in human and animal diseases. The rare event of its transmission from avian and mammalian reservoirs (mostly bats) to the human host has led to widespread epidemics in recent years (1). Indeed, over the last two decades, three CoV outbreaks have forced human populations to change their perspectives regarding the control of emerging diseases and the importance of public health in general.

The first outbreak caused by severe acute respiratory syndrome coronavirus 1 (SARS-CoV-1) occurred between November 2002 and July 2003, originating from China and then spreading worldwide (2). Although the symptoms of SARS-CoV-1 infection were in most cases non-specific, including lethargy, myalgia, and headache, the high mortality rate of 10% in case series was mostly related to respiratory failure due to acute respiratory distress syndrome (ARDS) (3, 4). The physiopathology underlying the severity of SARS-CoV-1 infection remained unclear after the epidemic due to insufficient sampling. A second CoV epidemic occurred in 2012 with Middle East respiratory syndrome (MERS)-CoV, which has mostly led to small-size outbreaks in the years ever since (5). Although it did not reach a pandemic status, MERS-CoV continues to infect humans, and the World Health Organization identified more than 850 patients who have died of related complications since its discovery (6). Indeed, MERS-CoV has a higher mortality rate in case series (case fatality rate of ~30%), mostly from respiratory failure, which has led to the identification of unique strategies of CoV infections to escape the immune response. Due to the ending of the SARS-CoV-1 epidemic and the somewhat limited number of cases of MERS-CoV in the recent years, understanding the mechanisms of CoV infections in humans has proven to be complex, and the conclusions drawn from *in vitro* experiments and animal models remain difficult to extrapolate.

In November 2019, cases of a pneumonia with atypical features were reported in Wuhan, China; in January 2020, SARS-CoV-2 was identified as the cause of this new CoV-induced disease (COVID-19), which became a worldwide pandemic in the following months (7). Although the mortality rates of this new COVID-19 are still being debated, ranging between 0.3 and 1.5%, it is still lower than those associated with SARS-CoV-1 and MERS-CoV infections. Patients suffering from severe SARS-CoV-2 infection could be healthy or only have mild comorbidities such as hypertension or diabetes (8). Most of all, severe cases due to respiratory failure occur 7–12 days after the first symptoms (9). Studies on COVID-19 have progressively stressed its similarities with previous CoV infections, mostly SARS-CoV-1, with the same unanswered questions regarding its physiopathology. One notable feature of this disease, already observed in previous CoV infections, is the high prevalence of obese patients among the most severe cases.

Here we seek to explore what underlies the link between immune response and respiratory failure in CoV infections on the one hand, and the current observation of obesity as a risk factor for severe outcome in COVID-19 on the other.

## Physiopathology of Respiratory Failure in Covid-19

Most of the time, the need for intensive care during COVID-19 is secondary to the onset of ARDS (9), as defined by the Berlin criteria (bilateral shadowing on lung radiology, rapid deterioration of symptoms, and objective hypoxemia on blood samples). In the first published series, 30% of these ARDS cases were accompanied by septic shock or other organ dysfunction (8, 10).

The nature of COVID-19-induced ARDS is still under discussion. Interleukin (IL) dosages are usually very high, and hypoxemia is severe in COVID-19-induced ARDS, which matches the hyperinflammatory profile described by Calfee et al. (11). SARS-CoV-1-induced ARDS was associated with vascular leakage and neutrophilic alveolitis (12), both of which are compatible with a hyper-inflammatory profile. In COVID-19, some experts observed ventilatory abnormalities suggestive of microcirculatory involvement such as hypoxic pulmonary vasoconstriction or distal thrombosis (13, 14). This points to the contribution of several factors in respiratory failure, with experts also citing the possible involvement of genuine viral pneumonia as well as capillary thrombosis by neutrophil extracellular traps (NETs) (15). The reason for this respiratory outcome is most likely a complex interplay of multiple factors, which derive directly from CoV virulence.

### Role of the Viral Gateway

The membrane protein angiotensin-converting enzyme 2 (ACE2) is used as an entry receptor by SARS-CoV-1 and SARS-CoV-2 (16, 17). It has been reported that SARS-CoV-2 has a greater affinity for ACE2 than SARS-CoV-1 due to the specific amino-acid composition in the receptor-binding domain of the spike protein (18). ACE2 is expressed at varying levels by most cells in the body but primarily in the small intestine, testis, kidney, heart, thyroid, and adipose tissue cells (19). The expression of ACE2 in adipocytes seems to be promoted by high fat diets (20). In the lungs, it is expressed by 2% of epithelial cells, increasing with cell differentiation, and it is mainly located on the apical (or luminal) pole, serving as an accessible anchor point to airborne contaminants (19).

ACE2 is a key enzyme of the renin-angiotensin system, converting angiotensin 2 (Ang2) into Ang1-7. Ang2 binds to a receptor, the angiotensin type 1 receptor (AT1R), a transmembrane G protein-coupled receptor, which is found in a large variety of cells, ranging from smooth muscle cells, endometrium, and myocardium to blood cells, renal interstitial, and glomeruli. The activation of AT1R has several effects: for example, vasoconstriction, vascular permeability, macrophage maturation, and pro-inflammatory cytokine release. During the resolution phase of the inflammation, Ang2 promotes tumor growth factor beta production and fibroblast proliferation, leading to fibrosis and inadequate healing of the wounded tissue (21).

An antagonistic pathway of the Ang2-derived effects results from the binding of Ang1-7 to the mitochondrial assembly (MAS) receptor. MAS receptor is a ubiquitous G-protein-coupled receptor, implicated, among others, in retina development (22), muscle wasting (23), and benign prostate hyperplasia (24). Activation of the MAS receptor by Ang1-7 induces vasodilatation by a nitric-oxide-dependent mechanism (25, 26) and reduces oxidative stress induced by Ang2 in vascular injuries (27). In macrophages, it promotes an anti-inflammatory profile (28), for example, by lowering pro-inflammatory cytokine production, notably IL-6 and tumor necrosis factor alpha (TNFα). Ang1-7 has also shown beneficial effects in inflammation resolution and fibrosis, notably in kidney and myocardial disease (21, 29). The binding of ACE2 by SARS-CoV-2 prevents it from exerting its enzymatic activities, resulting in decreased anti-inflammatory Ang1-7 production and the accumulation of pro-inflammatory Ang2 (16, 17). This results in high cytokine titers, neutrophil infiltration, and endothelial dysfunction in the lungs, potentially predisposing for ARDS.

As early as 2004, ACE2 tampering was suggested to be an important mechanism in SARS-CoV-1 infection (30, 31). It was only later discovered that CoV possesses very specific mechanisms to escape the host's immunity (32). These mechanisms, in addition to the pro-inflammatory response secondary to ACE2 binding, might act as a trigger for a sustained and uncontrolled inflammatory response, leading to ARDS.

### Immune Polarization and Its Consequences During CoV Infections

In general, an efficient antiviral response is driven by T-helper lymphocytes (LTh) with a specific polarization such as LTh1 and LTh2. LTh1 refers to a polarization in which LTh primarily promotes cytotoxic lymphocytes (CTL) and natural killers (NK) for the control and destruction of infected cells as well as the release of specific cytokines, such as type 1 interferon (INF-1) by innate immune cells.

INF-1 is produced by infected cells and innate immune cells after recognizing the viral pathogen-associated molecular patterns (PAMPS), such as single-strained or uncapped RNA, using cytoplasmic pattern-recognition receptors (PRR). In particular, toll-like receptor 3 (TLR3) induces Toll/interleukin-1 receptor domain-containing adapter-inducing interferon-β (TRIF). Hosts deficient in either TLR3 or TRIF are more susceptible to viral injuries and thus more at risk of developing ARDS during CoV infections (33).

INF-1 activates the Janus kinase-signal transducers and activators of transcription (JAK-STAT) pathway, resulting in the modulation of hundreds of interferon-sensitive genes and notably in the synthesis of specific cytokines, preferably oriented toward viral control and clearance (34).

Most of these steps, involved in INF-1 signaling, are blocked by CoV infections. This evolution trait is probably due to the presence of a constitutive INF-1 production in bats (principal reservoir of CoV). CoV infections are expert evaders of this antiviral response (35). Their escape plan revolves around three main mechanisms:

- First, hiding viral RNA from cytoplasmic PRR. After entering the cell, SARS-CoV-1 shields its RNA by forming, inside the host's endoplasmic reticulum, a large network of double-membrane vesicles isolated from the cytosol (36, 37). The modified capping of the viral RNA 2′-O-methylation also prevents the binding to an important cytosolic PRR (38).
- Next, direct tampering of the PRR-related enzymes. For example, the papain-like protein in CoV can modify the ubiquitinylation profile of TLR7 (39) or other antiviral-related PRR (40). Moreover, S protein triggers IL-1R-associated kinase and peroxisome proliferator-associated receptor gamma, subsequently downregulating interferon regulatory factor 7 activity (41). In addition, the jamming of TLR3 phosphorylation reduces the PRR activity, while blocking most of the INF-1 production pathways.
- Lastly, the non-structural protein 1 in both MERS and SARS-CoV-1 can selectively degrade host RNA via endonucleolytic activity against which the viral RNA is protected (42, 43).

The many mechanisms used by CoV probably leave the infected cells in a defensive cul-de-sac where they are incapable of developing an efficient antiviral response. On the one hand, viral PAMPS do not result in INF-1 production. On the other hand, non-viral PAMPS such as debris from cell lysis still stimulate the immune response. This could lead to inappropriate cytokine environments that lack INF-1 and are thus less effective against viruses, as seen in COVID-19 (44).

Indeed, during COVID-19 infection, most patients exhibit a specific cytokine profile, associating innate immunity chemokines (such as monocyte chemoattractant protein 3 and interferon gamma-induced protein 10 (IP-10), which are suggestive of macrophage activation and epithelial suffering), and pro-inflammatory macrophage-produced cytokines such as IL-6 (45). Moreover, CoV infections can directly induce the activation of nuclear factor kappa B (NFkB), notably by tampering with the TNF receptor-associated factor 3 pathway (TRAF3) via its open reading frame 3a. Activation by ubiquitination of TRAF3 also promotes the *de novo* development of the NOD-like receptor pyrin domain containing protein 3 (NLRP3) inflammasome and the production of IL-1β and IL-18 (46). This cytokine production promotes macrophage activation and INF-3, although it does not salvage a deficient polarization of the adaptive immunity toward LTh1 and its subsequent efficient antiviral response. High plasma levels of IL-6 and the absence of INF-1 have been noted in severe patients (47), illustrating a sustained innate response that fails to achieve viral clearance and triggers ARDS.

However, this sustained inflammation without LTh polarization might not be the only profile to bypass the antiviral cul-de-sac. Some patients infected by MERS-CoV demonstrated a polarization of the immune profile toward a LTh17-mediated response. Faure et al. compared two cases of MERS with different outcomes (48); the patient with a fatal outcome had an early increase in IL-17 and IL-23 titers (hallmarks of LTh17 polarization), whereas the surviving patient had a spike in INF-1 but no indication of LTh17 polarization. LTh17 are effective actors in the clearance of extracellular microorganisms such as fungi and bacteria, but poorly effective against viral pathogens (49). In general, viral PAMPS do not usually polarize the immune response to LTh17.

The association of severe outcome and inappropriate cytokine environment in CoV infection suggests a link with immune polarization, as a result of the “cul-de-sac” of antiviral response induced by the CoV escaping strategies. The resulting inefficient immune profile leads to a sustained viral exposure and persistent inflammatory state. In addition to the pro-inflammatory signals mediated by ACE2 inhibition, this sustained and inappropriate immune activation might be strongly involved in the development of ARDS.

## Implications of Covid-19 in Obese Patients

Obesity is a common condition, affecting up to 30% of adults in Western countries. It is defined by a body mass index (BMI) >30 kg/m^2^, irrespective of the location of the adipose tissue. However, all profiles of obesity are not equivalent in terms of their consequences. Indeed, abdominal (or visceral) obesity (estimated by the waist circumference or waist-to-hip ratio), in which visceral fat predominates, is more associated with metabolic disorders such as type 2 diabetes or hypertension, compared to “metabolically healthy” obesity, in which subcutaneous fat predominates.

**Figure 1:**
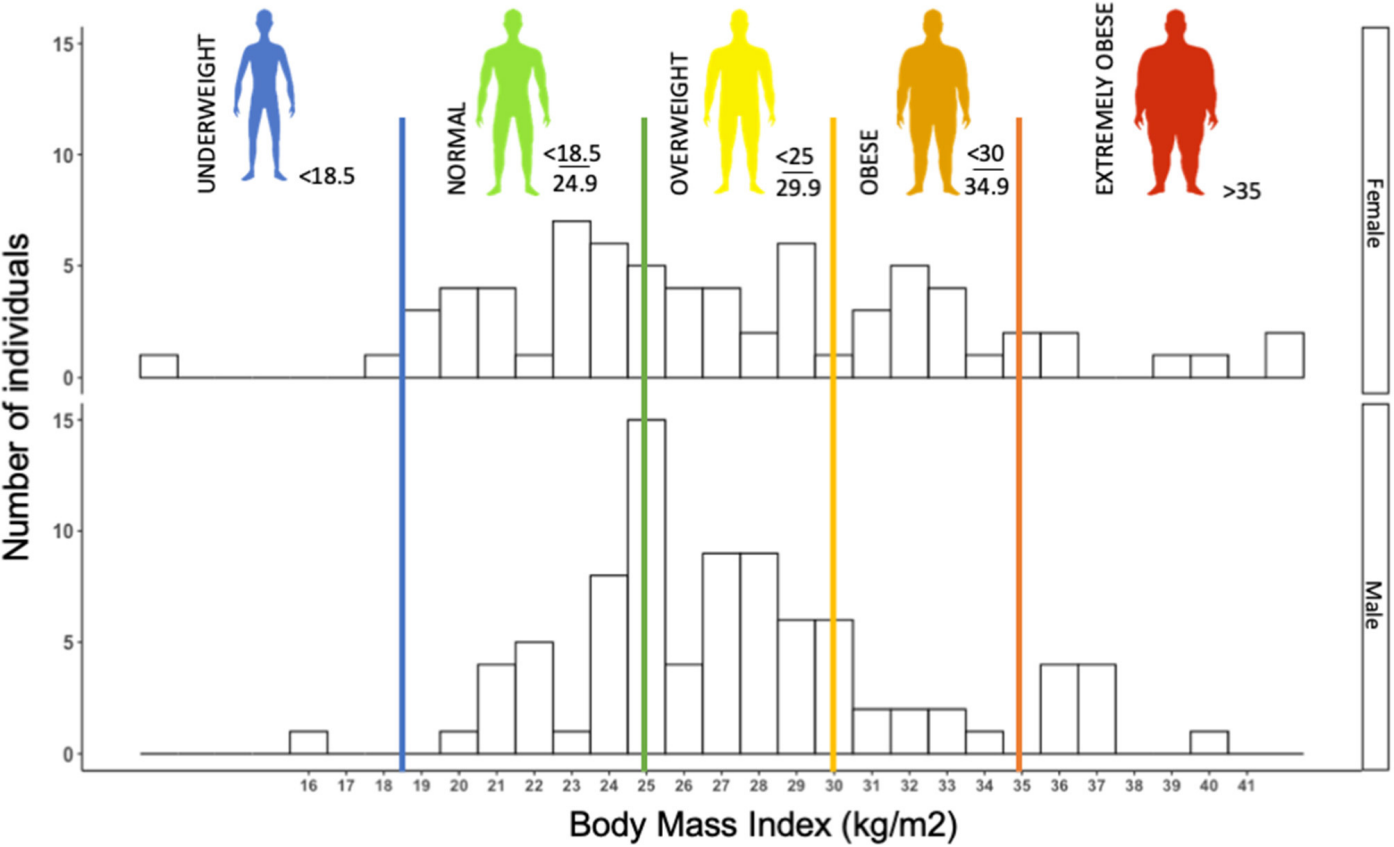
Histogram of the distribution of body mass index (BMI) (kg/m^2^) in 155 consecutive patients (female and male) admitted to Grenoble University Hospital for severe acute respiratory syndrome coronavirus 2 (SARS-CoV-2) infection (retrospective cohort study). Histogram illustrating that a majority of COVID patients, more precisely, 64% of COVID-19 patients were overweight or obese (BMI > 25 kg/m^2^). The median BMI of females (*F*) and males (*M*) was 26.30 and 27.08 kg/m^2^, respectively.

Early observations in the SARS-CoV-2 epidemics suggested obesity to be a risk factor to COVD-19, or at least to severe forms of the disease (50). In our retrospective cohort, we observed more than 60% of patients with overweight or obesity (*n* = 155) (Figure 1). In a retrospective cohort, Simonnet et al. showed an increasing risk of intensive care unit (ICU) admission in COVID-19 patients as BMI increased, independently of other metabolic disorders (51), which was subsequently confirmed by other teams (52, 53).

Thus, obesity appears to be a risk factor for presenting a severe form of COVID-19. It should be mentioned that once in ICU, obesity is known to confer a survival advantage, termed the “obesity paradox” (54). Patients with a BMI > 25 kg/m^2^ seem to survive mechanical ventilation and severe septic states significantly better than patients with a normal or low BMI (55, 56), presumably due to their elevated muscle mass, which represents a metabolic reserve in the hypermetabolic state of ICU patients (54, 57). It is not yet known whether once admitted to ICU, obese COVID-19 patients also present a better prognosis than patients of normal body weight.

### Adipokines

The scientific observations of the last two decades have placed obesity in a complex pathological framework centered around the deregulation of adipocyte, which is far from the naive idea of a simple diet-induced condition (58).

White adipose tissue (WAT) is now recognized as an independent endocrine organ, whose main role is to regulate and store the energy provided by food. However, the hormones released by WAT, specific to the adipocyte and known as adipokines, reach a large variety of organs and modulate an extensive range of functions, from appetite control to inflammatory response (29). Leptin is the leading adipokine, whose anorexigen properties regulate satiety and food intake. Leptin levels in blood are proportionate to the amount of WAT and increase with BMI. Interestingly, the leptin receptor (LEPR) on immune cells mostly activates JAK-STAT and NFkB dependent pathways, except in neutrophils, macrophages, and antigen presenting cells, which all express a particular form of LEPR. Leptin promotes migration in the WAT of resident macrophages and induces their polarization toward a pro-inflammatory profile or a classical activated macrophage (M1) profile, and unbalances the LTh profiles, by reducing regulatory T-cells and promoting LTh17 polarization (59). Adiponectin is another adipokine, whose levels increase in proportion to subcutaneous fat but decrease with visceral fat accumulation. It favors whole-body insulin sensitivity, fatty-acid oxidation and diminishes the hepatic neo-glucogenesis pathways (60). Adiponectin promotes primarily LTh1 polarization, hence antiviral inflammation. Other adipokines, such as lipocalin-2, down-regulate inflammatory LTh altogether by promoting regulatory lymphocytes. Adipokines form a large family regularly counting new members over the last few years, all of which reveal complex and multiple implications in the regulation of energy storage and release, adipose tissue regulation and rather ubiquitous cellular metabolism (61).

Unlike subcutaneous fat, visceral fat accumulation, also described as “abdominal obesity,” is characterized by a dysfunctional profile of adipokines associated with a rise in pro-inflammatory signals. The triggers of this dysfunction is believed to be a metabolic stress in the presence of nutrient excess and a hypoxic stress caused by hypertrophic visceral adipocytes, due to an increase in cells' size and low neovascularization, via a mobilization of Hypoxia Inducible Factor 1 (62). Unlike visceral fat, subcutaneous fat expansion is hyperplasic and is not correlated with low-grade inflammation (63). In severe abdominal obesity, the adipokine profile is unbalanced in favor of leptin production and low-grade inflammation at the expense of adiponectin, or lipocalin-2. This deregulation of the adipokine profile links various disorders associated with metabolic diseases, such as insulin-resistance, to inflammatory manifestations, as described in rheumatoid arthritis (64).

Ang1-7 takes an active role in regulating the effects of adipokines. Its involvement was reviewed by Lelis et al., with an exhaustive approach and emphasis on other adipokines that will not be described here, such as sirtuin and resistin (29). A strong interest in Ang1-7 has already arisen from these observations, particularly in the field of atherosclerosis and non-alcoholic fatty liver disease, in which Ang1-7 seems beneficial. In a concise article, Mori et al. hypothesized that the disruption of the renin-angiontensin system by the virus could impair the energetic functions of these pathways during SARS-CoV infections (65). We suggest that the tampering with such pathways could also lead to abnormalities in the inflammatory response observed in severe CoV infections through their influence on immune regulation and cytokine production.

### Meta-Inflammation in Obese Patients and Viral Response

Adipocyte dysfunction in visceral fat is correlated to low-grade persistent inflammation, known as meta-inflammation, which is suspected to be the starting point or an early factor in metabolic disorders associated with severe obesity (63). This meta-inflammation is mostly driven by the leptin-activated M1 macrophages in WAT. WAT-resident macrophages exhibit pro-inflammatory behavior, producing IL-1β, IL-6, and TNFα. The precursor of IL-1β is cleaved into bioactive IL-1β by the NLRP3 inflammasome, as a result of the NFkB pathway activation, which is induced by both pro-inflammatory and hypoxic signals originating from the adipocytes (58). Adiponectin can inhibit NFkB activation, but as mentioned above, depending on the obesity severity and profile, the effects of adiponectin can easily be overwhelmed by those of leptin (66).

Leptin also polarizes hematopoiesis directly in the bone marrow, promoting granulocyte, and erythroblast lines (the latter probably acts as a protective mechanism against hypoxia) at the expense of lymphocytes (67). When neutrophils are mature and circulating, leptin also promotes their survival on a dose-dependent scale (68). Higher levels of neutrophils have thus been observed in obese patients, possibly making the neutrophil recruitment during an inflammatory process more potent than in patients with a normal BMI (69).

Besides suffering from a pro-inflammatory environment, which favors macrophage activation and neutrophil production, obese patients exhibit abnormal responses to viral infection. As summarized by Honce et al., during influenza infections, obese patients tend to have greater neutrophil activation and NET development, contributing to capillary damage and thrombosis. Such phenomena have been extensively found in COVID-19 patients (70). Their inflammatory response is also characterized by a lack of INF-1 production as well as a strong cytokine production, notably IL-6, IP-10, and type 3 INF, which are elevated in severe COVID-19. Interestingly, patients with visceral fat accumulation also tend to have a lower TLR3 expression in adipocytes, muscle cells, and adipose tissue-resident macrophages, as well as a concomitant lower production of cytokines following exposure to viral PAMPS (71–73). This suggests that their baseline profile resembles that found in severe CoV infections, in which the antiviral response is less efficient, but the overall inflammation is higher than in other viral infections.

Finally, both obesity and metabolic disorders are associated with vascular dysfunction. At the acute phase of lung infection, this could result in microcirculatory abnormalities, as suggested by intensive care physicians, and increased lung edema.

## Discussion

Patients with visceral fat accumulation, type 2 diabetes (74), and hypertension are not the only subjects at a higher risk of severe SARS-CoV-2 infection. When considering metabolic disorders separately, diabetes, non-alcoholic liver disease, and obstructive sleep disorders have been recently reported as risk factors for a severe outcome (74–76). This suggests that the metabolic dysfunction associated with these disorders more than obesity alone might be involved in the severity of the disease in these patients.

**Figure 2:**
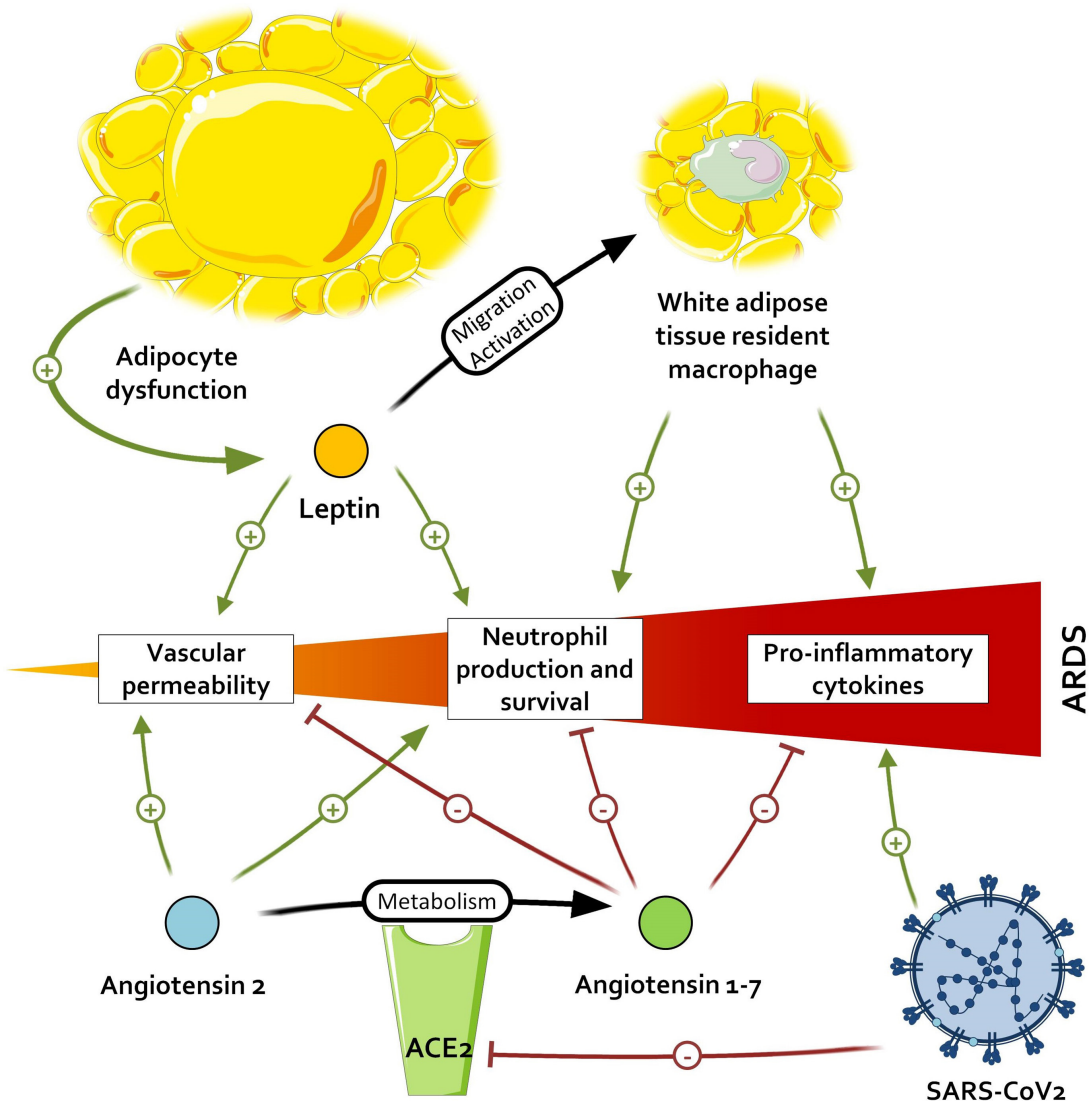
Impact of severe acute respiratory syndrome coronavirus 2 (SARS-CoV-2) on pathways promoting acute respiratory distress syndrome (ARDS). By inactivating the angiotensin conversion enzyme 2 (ACE2), SARS-CoV-2 leads to an accumulation of angiotensin 2 and a lower dosage of angiotensin 1-7, respectively resulting in the higher promotion and lower inhibition of pro-inflammatory signals.

When comparing the effects of Ang1-7 and the inflammatory environment of patients with adipocyte dysregulation and metabolic disorders, an interesting pattern emerges. All the immunological features arising from the adipocyte dysfunction—(i.e., M1 macrophage polarization with IL-6 and TNFα production), and neutrophil promotion—may contribute to the development of ARDS and thus be countered by the activation of the Ang1-7/MAS receptor axis. Ang1-7 also favors a strong capillary barrier and a beneficial oxidative profile, which are altered in patients with visceral fat activation and could help to prevent ARDS. This leads us to two hypotheses: either patients with metabolic disorders, primarily visceral fat accumulation, have a constitutional lower titer of Ang1-7, as suggested by some observations (77), and a resulting higher inflammation; or the Ang1-7 levels in these patients are preserved and restrain the baseline inflammation. In the first case, the inappropriate inflammatory response, added to the diminished activation of TLR3 in obese patients, leads to unrestrained inflammation. However, if Ang1-7 is present in these patients and limits the meta-inflammation, acting as a guardrail, the antagonization of ACE2 by SARS-CoV-1 and 2 in addition to the lack of *de novo* Ang1-7 production could exacerbate the meta-inflammation and contribute to the severe septic states of obese patients with COVID-19, as illustrated in Figure 2. In both cases, the supplementation of Ang1-7 in these patients might improve fitness upon SARS-CoV infection.

ACE2 deficiency has already been explored by some research teams to better understand the potential metabolic benefits of conversion enzyme inhibitors used in hypertension, among others. Their studies highlighted the association between ACE2 deficiency and higher titers of pro-inflammatory cytokines in obese mice, as well as in mice with glucose intolerance (78), which is closely correlated with meta-inflammation (79). Other studies correlate ACE2 deficiency with epicardial inflammation (80). This suggests that the Ang1-7/MAS axis allows a better control of inflammation in obese patients.

TLR4 is a receptor to LPS and leads to NFkB activation and (among others) hepatic inflammation. When administered orally to rats fed with a high-fat diet, Ang1-7 lowered hepatic inflammation, notably through a modulation of a metabolic pathway involving TLR4 (81). Moreover, promoting the effects of the Ang1-7/MAS receptor axis using medication also improves the aforementioned cytokines and oxidative stress in obese mice, with a protective effect against diabetic cardiomyopathy (82).

Ang1-7 is already in the spotlight of scientific research given its beneficial effects in preventing the development of metabolic disorders and obesity (83). We believe that our literature review highlights the beneficial effects of Ang1-7 on meta-inflammation in preexisting obesity and its potential involvement in inflammatory response and viral clearance, notably against SARS-CoV-2. Modulation of the renin-angiotensin system has been mentioned by others to explain the severity of COVID-19. A recent study found a lower mortality and intubation risk during COVID-19 among elderly patients treated with nifedipine or amlodipine (84), although the study sample was small and most of the accessible data do not suggest a strong connection (85, 86). However, these drugs interfere with AT1R and not with the genuine production of Ang1-7.

In obese patients with COVID-19, this hypothesis should be considered. Oral or parenteral Ang1-7 supplementation could be a therapeutic option to diminish the low-grade systemic inflammation due to adipocyte dysfunction and attenuate the severity of ACE2-mediated injuries consecutive to SARS infection. Parenteral Ang1-7 has already been used in human research on account of its property to enhance acetylcholine-mediated vasodilatation in endothelia, with safe outcomes (87).

## Conclusion

COVID-19 is a viral disease with remarkable characteristics given its high severity in obese patients and its ability to tamper ACE2 metabolism. We believe that more than being just an incentive to accelerate research on viral infection, COVID-19 also presents an opportunity to respond to questions that were previously considered to be too intricate or complex, such as non-septic inflammation or the immune system communication underlying metabolic disorders. Understanding the multiple and interrelated factors linking SARS-CoV-2 infection, angiotensin metabolism, global inflammation, and metabolic disorders such as type 2 diabetes and obesity should provide us with a better insight into the way in which these conditions and physiological states interact outside of an acute aggression.

## Data Availability Statement

The raw data supporting the conclusions of this article will be made available by the authors within respect of General Data Protection Regulation, without undue reservation.

## Ethics Statement

The studies involving human participants were reviewed and approved by Comité Ethique du Centre Investigation clinique (CECIC). Subjects were all informed and did not oppose, written consent for participation was not required for this study in accordance with the national legislation and the institutional requirements.

## Author Contributions

GM and AL conceptualized the idea, provided discussion, feedback, and organize the plan of the article. GM, A-LB, OE, and AL wrote the paper. GM, A-LB, OE, BT, and AL reviewed the different version of paper. All authors contributed to the article and approved the submitted version.

## Conflict of Interest

The authors declare that the research was conducted in the absence of any commercial or financial relationships that could be construed as a potential conflict of interest.
